# Supplementary material for: Womb to womb: Maternal litter size and birth weight but not adult characteristics predict early neonatal death of offspring in the common marmoset monkey
Source: PLoS One. 2021 Jun 9;16(6):e0252093. doi: 10.1371/journal.pone.0252093 (PMC8189522; doi:10.1371/journal.pone.0252093)
Supplement: Attachment — Submitted filename: Rutherfordetal_PerinatalMortality_Response_Rev2.docx [file pone.0252093.s002.docx]

Response to reviewers

We are delighted by this second review of our manuscript. Thank you so much for such positive, constructive feedback throughout this process. We made the three changes suggested by Reviewer #1, outlined below:

Reviewer #1 (line numbers correspond to marked manuscript):

- Line 42: We changed “higher birth weights” to “higher birth weight.”
- Line 60: We substituted the phrase “omit, combine, or obscure” for “elide or obscure.”
- Line 161 (actually line 261 in the unmarked manuscript, 262 in the marked manuscript): We changed “thought” to “though.”

We will be making our data publicly available as soon as possible.

Sincerely,

Julienne Rutherford, on behalf of all of my co-authors
